# Obstetrics and gynecology applicant perceptions of residency program culture with virtual interviews: a qualitative analysis of social media posts

**DOI:** 10.1186/s12909-024-05175-x

**Published:** 2024-03-08

**Authors:** Laura H Jacques, Elise S. Cowley, Shanaya M. Hebgen, Ryan J. Spencer, Corinne M. Hale

**Affiliations:** 1 Department of Obstetrics and Gynecology, School of Medicine and Public Health, University of Wisconsin-Madison, McConnell Hall, 4th Floor, 1010 Mound St, 53715 Madison, WI USA; 2 Department of Bacteriology, University of Wisconsin-Madison, 1550 Linden Dr, 53706 Madison, WI USA; 3 Microbiology Doctoral Training Program, University of Wisconsin-Madison, 1550 Linden Dr, 53706 Madison, WI USA; 4 Doctor of Medicine program, University of Wisconsin School of Medicine and Public Health, 750 Highland Ave, 53726 Madison, WI USA; 5 Department of Anthropology, University of Wisconsin-Madison, 1180 Observatory Dr, 53706 Madison, WI USA

**Keywords:** Residency interviews, Obstetrics and gynecology, Virtual interviews, Program Culture, Applicant perceptions, Residency Program Fit

## Abstract

**Background:**

In the United States, Obstetrics and Gynecology residency interviews are instrumental in assessing the compatibility between medical student applicants and residency programs during the match process. Applicant perceptions of Obstetrics and Gynecology residency culture are a key component in determining how they rank residency programs. In 2020, residency interviews transitioned to a virtual format, and little is known about how applicants evaluated program culture during this first round of universal virtual interviews. Medical students in the United States commonly use Reddit, a popular social media platform, to discuss residency programs and share interview experiences. We explored Obstetrics and Gynecology applicants’ considerations regarding residency program culture during the first universal virtual interview season in 2020–2021 by analyzing posts on a Google spreadsheet accessed through Reddit.

**Methods:**

In 2022, we imported 731 posts from the “2020-21 OB GYN Residency Applicant Spreadsheet” Google spreadsheet posted to the 2020–2021 Residency Interview Spreadsheet megathread on the r/medicalschool subreddit to NVivo 12(QSR International, Burlington, MA), a qualitative analysis software program. Three investigators used qualitative inductive techniques to code and identify themes.

**Results:**

Applicants used visual, verbal and behavioral cues during virtual Obstetrics and Gynecology residency interviews to understand three components of the workplace culture: prioritization of diversity, equity and inclusion, social environment, and resident workload.

**Conclusions:**

Obstetrics and Gynecology residency programs convey information about their culture during virtual interviews through the behavior, appearances and responses of residents and interviewers to applicant questions. To ensure they accurately represent their culture to applicants, programs should consider educating residents and faculty around the implications of interview-day conduct.

## Introduction

When medical students in the United States (US) apply for Obstetrics and Gynecology (ObGyn) residencies, they take into account various factors, including location and institutional reputation. However, consistently ranking as a top priority for both applicants and programs is the interview day experience and residency program culture [1–6]. Residency interviews serve as a critical way for applicants to assess residency program culture, influencing how students ultimately rank programs during the Match® process [2, 3]. The onset of the COVID-19 pandemic in the US forced the 2020–2021 interview season to transition online to combat the spread of SARS-CoV2. Ultimately, even after the threat from COVID-19 diminished, the Association of American Medical Colleges (AAMC) and the National Resident Matching Program (NRMP) recommended the adoption of virtual interviewing for all residency programs to address socioeconomic disparities, reduce environmental impact, and enhance applicant satisfaction [7, 8]. Though virtual interviewing conveys many advantages, both applicants and program directors have found it difficult to convey and assess program culture, especially when compared to traditional in-person interviewing [1, 5, 9]. 

If programs want to accurately convey their culture to applicants, they must understand which aspects applicants value and how they gather this information virtually. There is little data on how applicants evaluate program culture during virtual interviews. We sought to evaluate what aspects of ObGyn residency program culture applicants prioritize and how they assess program culture during virtual interviews by analyzing posts to a Google spreadsheet on Reddit, a popular social media platform.

## Materials and methods

### Data source

Reddit, a popular social media platform in the United States, counts nearly 40% of people 18–29 years of age as users, and functions as an interactive online bulletin board [10, 11]. Users post content and engage through narrative comments within topic-specific communities called “subreddits” which contain discussion boards known as threads. Reddit is increasingly used as a qualitative data source due to its ability to capture real-time, often unfiltered impressions and discussions of events [12–17]. 

Every year the subreddit /r/medicalschool creates a publicly available “megathread”, a consolidated thread used for major events or popular topics within a subreddit, where medical students discuss residency programs. Specialty-specific spreadsheets are posted within the megathread. On May 27, 2022, we accessed the “2020-21 OB GYN Residency Applicant Spreadsheet” Google spreadsheet posted to the 2020–2021 “Residency Interview Spreadsheet” megathread on the r/medicalschool subreddit. We chose this time frame because it represented the first interview season where there was widespread use of virtual interviewing. We analyzed every comment from the three tabs most pertinent to our research question - “Name & Shame”, “Student Reviews,” and “PM_Pearls”. The ‘Name and Shame’ tab features student reviews detailing their interview experiences with specific residency programs. In this context, ‘Name’ refers to students disclosing the program they interviewed with, while ‘Shame’ pertains to any issues, comments, or activities by the programs or their representatives that caused applicants distress or frustration. “Student reviews” is a tab where students evaluate their home institution and respond to questions about the experience at their program from other students. “PM_Pearls” is a section where applicants and program managers can engage in discussions and address applicant questions and concerns.

A single researcher removed duplicate posts and identifying information like usernames and location. We imported 731 unique anonymous posts to NVivo 12(QSR International, Burlington, MA), a qualitative analysis software. While each post had a unique username, there is no way to verify that each username came from a different individual.

### Analysis

We analyzed 731 poster comments using thematic qualitative analysis, as described by Kim and colleagues [18]. Three investigators, C.H., E.S.C. and S.H., used an inductive approach to generate codes based on the data and create a codebook. The final codebook contained 11 codes and 38 subcodes. The first 25 posts were collectively coded by C.H., E.S.C., and S.H. to reach a consensus, after which the investigators individually coded all remaining posts. Three of the authors met weekly to discuss coding and compare how each coder applied codes to the content to ensure the researchers coded consistently and in alignment with each other. These meetings were reflexive exercises to address different approaches to coding and reach consensus around when to apply specific codes. All five authors independently reviewed the coding reports, met together as a group to discuss and generate the final three themes presented below. This study is considered program evaluation and was exempted from review by the UW-Madison Institutional Review Board.

### Positionality

It is important to consider one’s own identity as it relates to the research topic, as this may bring bias into the interpretation of the data. At the time this data was analyzed, L.J. held the position as the Obstetrics and Gynecology medical student clerkship director and R.S. was the director of the Obstetrics and Gynecology residency program for the University of Wisconsin Madison. C.H. was a doctoral student in the department of Anthropology, E.C. was an M.D. PhD student in the doctoral portion of her training and S.H. was a second-year medical student. While our academic roles contribute to our deeper understanding of the subject matter, it is important to acknowledge our backgrounds as it may relate to our research process and outcomes. Two of the authors are faculty in roles that require them to engage with medical students and residents, which could have influenced their understanding of the commentary analyzed. Three of the authors are students and two of those students are preparing to enter residency programs in years to come. As students, they bring unique perspectives and recent lived experiences within medical education spaces to this research at the time of analysis.

## Results

**Fig. 1:**
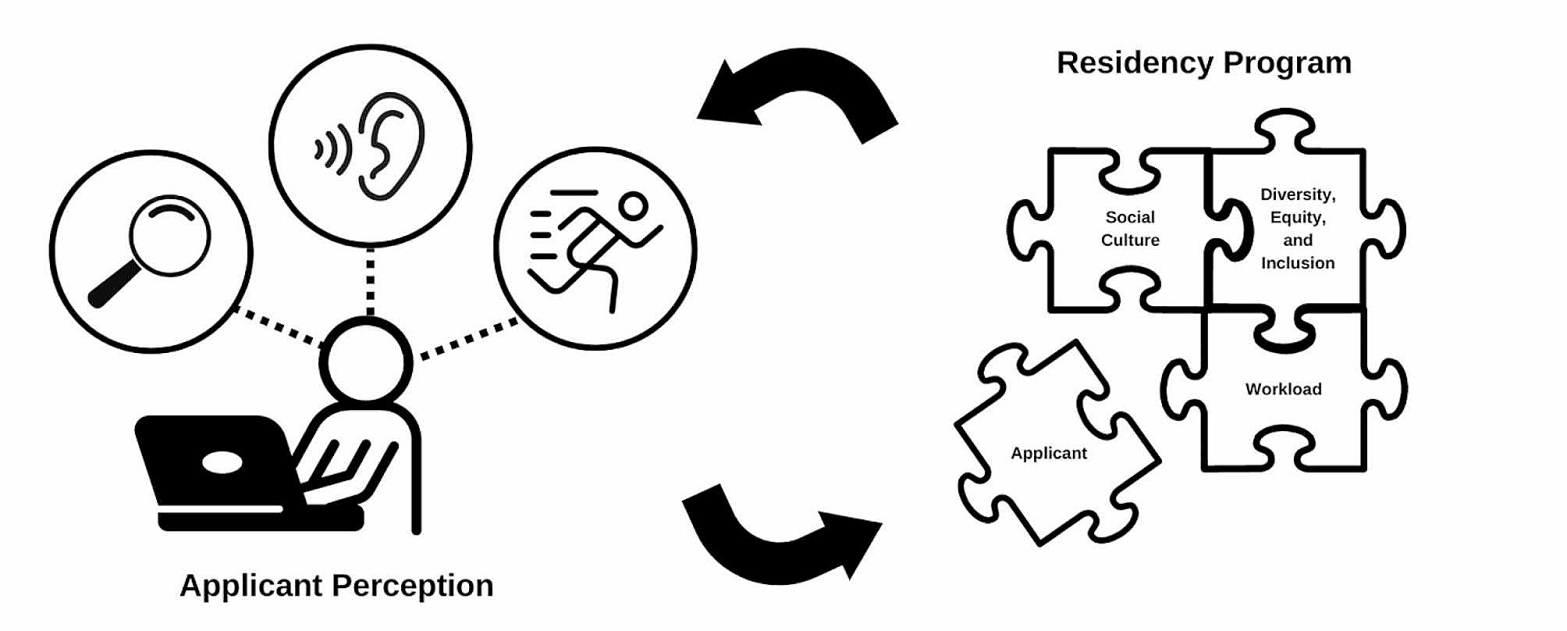
*How obstetrics and gynecology residency applicants evaluate program culture during virtual interviews*. Obstetrics and Gynecology residency applicants use *visual, verbal*, and *behavioral* cues to evaluate three components of residency program culture, social culture, DEI, and resident workload and burnout, during virtual interviews

We identified three themes that relate to what applicants value in a residency program’s culture: (1) Prioritization of diversity, equity, and inclusion (DEI), (2) Social environment and resident (mis)treatment, and (3) Resident workload. The key themes were identified based on frequency of codes and subcodes the researchers applied to the 731 comments analyzed to identify each theme. Comments related to the social environment and resident mistreatment in programs appeared most frequently, followed by comments related to resident workload, then prioritization of DEI. These themes captured the majority of the focal points presented in the comments. Comments included other areas of potential interest, including accessibility concerns, but these comments were limited and outside of the scope of this paper. Our analysis also revealed three coherent subthemes for each main theme regarding *how* applicants were trying to determine these three aspects of residency culture during virtual interviews; they used (a) *visual, (b) verbal* and (c) *behavioral* clues to understand program culture (Fig. 1).

### Prioritization of diversity, equity and inclusion

Applicants were keenly interested in how programs were addressing issues around diversity, equity and inclusion (DEI) during the 2020–2021 application cycle [19, 20]. This time period in the US was marked by significant cultural events like the tragic death of George Floyd and COVID-19 driven health disparities that thrust matters of social justice and racial inequality into the spotlight [20, 21]. Posters commented on the varying sources, visual, verbal and behavioral, they used to try to determine how residency programs were handling issues of race, equity and inclusion. This theme was so prevalent in our data, that we dedicated a separate manuscript to exploring this theme and the impact of DEI on the residency match process [19]. We also deemed it essential to include a more concise exploration of the DEI theme in this paper, as it emerged as a key factor in applicants’ assessments of program culture and played a significant role in shaping their perspectives during the residency selection process.

#### Visual cues

Applicants considered the visual diversity among interviewers, current residents and applicants as an indicator of inclusivity. For instance, one post mentioned, *“Was disappointed by lack of diversity in applicant pool during my IV [interview] day”.* Several other posters agreed with one replying, *“yup especially given they have ZERO black residents right now”*. One applicant expressed disappointment with the lack of perceived diversity at an event specifically designed for under-represented in medicine applicants, “*Had us attend a 6-hour diversity 2nd look with ZERO black or Latinx (from appearances and names) applicants present. Idk [I don’t know] what they’re doing but that was my sign.”*

#### Verbal cues

Verbal cues about the inclusivity of program culture included how interviewers asked and responded to diversity, equity and inclusion (DEI) focused questions, microaggressions, and culturally insensitive or racist comments made by members of the department. One student of color described being asked an inappropriate question by an associate program director, *“I was asked what I do to be anti-racist by the APD [Associate Program Director]. I’m Black so that was super off putting and it was a very poor question to ask a Black woman.”* Others empathized with that student’s experience and reaffirmed their assertion that it was a racially motivated question, *“YIKES. I was also not asked this (and I’m white!)”.* Applicants also posted about experiencing microaggressions during interviews such as “*Question on diversity efforts was met with how they look for “competent” applicants, WTF”* and “*Of course got the “Your English (sic.) so good” thrown in by half my interviewers. Just a ton of microaggressions I have neither the energy or interest in repeating”*. Several posters described racist comments made by interviewers such as a program chair who referred to undocumented people as “*illegal immigrants*” or residents characterizing the community where they live as “*the hood*”, which they found “*absolutely appalling”* and *“blatantly racist.”*

#### Behavioral cues

Applicants also evaluated program culture based on the behavior of programs around DEI-related issues, such as what faculty posted on their private social media accounts and whether institutions have faced discrimination-related litigation. Posts range from critical, *“Associate Dean of GME ([Institution name]) has been sued THREE TIMES for discrimination, yet still retains his position… definitely sensing a systemic problem here.”* to noting positive actions like hiring more female faculty and increasingly diversifying their resident classes.

### Social environment and resident (mis)treatment

Applicants actively discussed their perceptions of the social dynamics within residency programs, emphasizing their interest in residents’ interactions and how they were treated by faculty and the institution. Visual, verbal and behavioral cues played a crucial role in applicants’ assessments of the social environment within the residency community and between faculty and residents.

#### Visual cues

Applicants noted that the presence or absence of residents from interview days visually signaled to them whether the program valued its residents. One poster registered “*No residents present during IV [interview] day*” as a “Shame” and another poster agreed replying, *“I think it is a huge red flag to not have residents present at an interview day and understand why op [original post] would state this*.”

*Verbal cues*: Verbal cues included the interviewing style, degree of interviewer interest in applicants and types of questions asked by interviewers to determine the social environment. Interrogative or behavioral interviewing were viewed the most negatively as they seemed too intense or didn’t facilitate programs and applicants to get to know each other. An exemplar post regarding an applicant’s perspective on a more interrogatory interview: *The interviews were like oral exams where we were asked obgyn questions- if you have a 15 min interview you should use that time to get to know us personally, not test us on info that we clearly were tested on in the usmle exams*.” Behavioral interviewing was also widely seen as a negative experience as exemplified by the following highly-engaged post (+ 4 – indicating four additional posters agreed with the preceding comment) and additional comments (<) expressed agreement:

Wanted to love this program. Seems like a great place and the residents were really nice, but my interview consisted of purely behavioral questions asking me to describe times where I messed up. Spent the whole day talking about every bad experience I’ve ever had and at the end of the day I just felt like shit. Can’t rank them high cuz (sic.) I just had such a terrible interview experience + 4 < Agree, I felt like they did not get to know me at all. They also didn’t respond after I did, so I told them a horror story and then they just stared at me and asked the next question… over and over.

The types of questions interviewers asked applicants also influenced their perception of program culture with many posters “shaming” programs for asking “illegal questions” about marital status, relationships and what other programs applicants were applying to. As one poster states, *“Illegal questions all over. PD asked me “so now that we are talking, tell me the real reason why you applied to this program” weird way to ask that question.”*

#### Behavioral cues

Applicants gauged a program’s attitude toward its residents and what the social environment was like by observing behavioral cues, including how residents were prioritized for COVID-19 vaccines, interviewer conduct and the behavior of residents during pre-interview social events. The interview cycle included in our analysis was the first during the COVID-19 pandemic, and prioritization (or lack thereof) of residents for the initial COVID-19 vaccinations was seen as an indicator of how the program valued residents. A representative post states, *“Sacrificing their residents to COVID by not prioritizing them for vaccination. Institutions treats their residents poorly, clearly. Top program so clearly think they can get away with this crap. Don’t rank friends.”* A popular post with applicants discussed the positive and negative ways residency programs were handling COVID-19 vaccine distribution in the early stages of the pandemic and what this meant about the way the program and the hospital system valued residents.

*“Saw on Twitter that the PD supported all the obgyn residents protesting… they arranged for L&D coverage from attendings/APPs so that residents could protest and it wouldn’t affect patient care > > yeah, but where were the admin in advocating for the residents prior to that? why were they not in the relevant rooms, or if they were, why were their voices not prioritized? >> yep exactly, love that the program leadership is supportive but it’s going to be a long 4 years if your INSTITUTION leadership treats residents like cattle. << Agreed. I know people want to*.

*simp* 1 *because it’s[Institution name] but this is a huge red flag as to the way institutions treat residents and I’m glad that at least covid is highlighting this at multiple places.”*

Disrespectful interviewer conduct was also commonly discussed by applicants. Students found interviewers arriving late or leaving early, not reading application materials, having webcams turned off or multi-tasking during the interview with the sense that faculty do not value trainees. A representative post reads:

The residents were really kind and I enjoyed my interview experience other than that but the faculty seemed SO uninterested in interviewing me. I interviewed with the interim PD, APD and 2 other faculty members. Some of the faculty had their cameras turned off and the APD answered a phone call when I was mid-sentence and left and never came back.

Another poster describes a negative interaction with a program director,

PD was chomping away on baby carrots during my interview, unmuted. And she decided to try to fix her internet connection issues during my interview (about halfway through the day) instead of during the break time. Overall got the sense she was uninterested.

One post more explicitly states how the interviewer’s behavior was viewed as a surrogate for what it would be like to work with them, *“One of my interviewers clearly didn’t read my application. I would not want to work with that faculty member.”*

Applicants commonly used pre-interview social events to observe the social culture of a program focusing on residents’ enthusiasm and friendliness. Notably, impressions of socials were often mixed, even from applicants who attended the same events. A representative post states, *“The residents seemed really hostile towards each other, especially the upper years. Did anyone else experience this?”* and another replied, *“not at all! I had the opposite impression. I loved the interactions between the residents, thought they got along incredibly well. It’s one of the biggest things that stood out to me about the program”*.

### Resident workload

Applicants wanted to find programs that offered high-quality training while also providing sufficient support for maintaining a work-life balance. They assessed the workload of residents at each program, seeking to strike a balance between rigorous training and personal well-being. Visual, verbal, and behavioral cues observed during virtual interviews and related social events were leveraged by applicants to gauge these aspects of residency program culture.

*Visual cues*: Applicants assumed residents were overworked if they appeared tired or continually mentioned how hard they worked, as seen in this post, *“Kept repeating how busy of a program they are. Residents seemed tired at meet and greet. Overall sense that they are overworked here.”* Another post describes the appearance of the interns at a pre-interview social event and then a medical student from that institution confirms the impression the residents are overworked *“Interns looked a little rough at the meet and greet lol, they all seemed so exhausted:/ << As somebody that goes to [Med School Name], they definitely work a lot. Its 12 + hours in all services except outpatient.”*

#### Verbal cues

Verbal cues included questions from interviewers about how applicants intended to balance the responsibilities of family and pet-ownership while being a resident, implying a lack of program support for residents’ personal lives. A representative post states, *“I also had some very uncomfortable questions like “well what are you going to do with your dog all day while you’re at work?” And “does your husband understand how much you’re going to be working?” (in a condescending, unfriendly tone).”*

#### Behavioral cues

Criticism of interview interruptions by pets or children were seen as evidence that the program may not understand the needs of its members as described in this post, *“During the social the residents multiple times made a very big deal about how unprofessional it was to have a dog or a partner around while you are interviewing. Like multiple times they were like “we take note if this happens and it looks bad.” Like yall chill it’s a damn pandemic and some of us live in studio apartments and can’t pay for pet care etc. Just rude.”*

## Discussion

We found that in 2020–2021, US applicants to ObGyn residency programs posting on the 2020-21 OB GYN Residency Applicant Spreadsheet on Reddit, prioritized diversity and a supportive social environment within a residency program and were wary of signs of resident mistreatment, excessive workload and burnout. Consistent with our findings, the 2022 NRMP applicant survey revealed that applicants rated “Overall goodness of fit” as their top priority and “Interview day experience” and “Work/life balance” as the second and fourth priorities when ranking programs (“Desired geographic location” was third) [1]. While there is no universally agreed-upon definition for “goodness of fit”, it is generally understood to rely heavily on the cultural alignment between program and applicant [6]. 

Historically, the NRMP applicant surveys were conducted every two years on odd years, so data for the 2020–2021 applicant cycle is not available. However, in 2022 the NRMP released data from 2021 to 2022 and compiled a research brief summarizing how the COVID-19 pandemic and the transition to virtual interviews has impacted programs and applicants. The survey results indicate that the majority of applicants encountered challenges in assessing program culture and gauging program commitment to diversity within the virtual environment. Applicants seemed to adapt to the virtual environment over time however and reported increased comfort assessing residency culture and commitment to diversity in 2022 versus 2021 [1]. Our data provides additional context to the NRMP data. The qualitative nature of our study allows for a more detailed description of the specific components of residency program culture that applicants value, as well as how applicants are adapting to the virtual environment and accessing this information online. Similar to applicants, the corresponding 2022 program director NRMP survey found that the majority of US program directors felt that the virtual platform disadvantaged them in showcasing their programs and finding aligned applicants [5]. Our data challenges this assumption and suggests that programs *are* conveying their program culture virtually, but perhaps in ways program directors have not considered. Programs can leverage our findings to inform improvements to interview processes so that both programs and applicants are able to effectively assess compatibility.

Many applicants in the 2020–2021 interview season reflected on issues of racism and equity within residency programs. It is possible the convergence of the growing Black Lives Matter Movement and the stark racial and health inequities exacerbated by the COVID-19 pandemic influenced applicants’ focus on these topics. However, racism and inequity have always existed in healthcare and applicants’ concerns in these areas will likely persist in future interview cycles. Residency programs must proactively address concerns and ensure they prioritize diversity and inclusion, particularly in selection of interviewers and interviewees. Programs should also be poised to actively engage in discussions around other pressing societal issues, including abortion rights and access as well as the burgeoning antisemitism and humanitarian crisis following the Israeli-Palestinian conflict. Programs can explain their strategies and steps taken toward addressing current issues and subsequent impacts on resident training and patient care.

Residency programs should ensure resident involvement in the interview process and make sure their residents appear, and are, well-rested prior to interview days and social events. Programs could consider faculty development around respectful interview behavior and reflect on how interview styles, such as behavioral or interrogative interviewing, convey their residency culture. Effective virtual communication requires distinct strategies, and it could benefit programs to offer guidance to interviewers in this regard [22–24]. Over time, as both applicants and programs become more adept at projecting themselves virtually, we anticipate applicants and program directors will gain greater confidence in assessing compatibility through virtual means.

While cultural alignment between a program and applicant continues to be a priority for both applicants and programs, it is important to remember that there is no evidence that this alignment results in better outcomes and focusing on cultural compatibility may have negative consequences. Cultural compatibility may be defined as similarity and can be a vehicle for unconscious bias perpetuating inequities and limiting diversity within programs [6]. Further research should evaluate whether achieving program and applicant alignment results in positive outcomes like improved resident workplace satisfaction or lower rates of resident burnout or generates negative outcomes such as reduced racial, ethnic and socioeconomic diversity within residency programs.

The strengths of our research are the qualitative nature, which allows for a more in-depth exploration of applicant considerations around program culture. Additionally, the anonymous nature of the posts gives a different, and perhaps less filtered, perspective of the applicant experience than traditional interview or survey data. As people are typically posting in real time, shortly after their interview experiences, recall bias is mitigated. While our study has strengths and provides valuable data, it is important to acknowledge the inherent limitations associated with using social media as a data source [25]. There is selection bias, as the data collected from Reddit represents the perspectives of applicants who chose to post their experiences on social media, which may not be representative of all applicants. Additionally, there is social desirability bias, where posts may not accurately reflect a user’s experience, but their desire to fit in or portray themselves in a certain way online. Importantly, there is also no way to verify the veracity of posts or an accurate number of posters, as one individual may post under several different usernames. Additionally, the data in this study is drawn from only one year, the 2020-21 interview cycle, which was a unique year as it was at the beginning of the COVID-19 pandemic and was the first time there was widespread use of virtual residency interviewing. These factors may limit the generalizability of the data.

## Conclusions

Our work offers valuable insights into the considerations of US ObGyn applicants when evaluating program culture during virtual interviews. We found that applicants were interested in the overall social environment of the program and how programs and departments prioritized DEI and resident well-being. Residency programs can benefit from understanding how their selection of interviewers, interview styles, and resident and faculty conduct contribute to conveying these aspects of program culture to applicants. While our research specifically focuses on applicants to ObGyn residencies, the findings likely hold relevance for applicants across specialties.

## Data Availability

The database for this project is publicly available on Reddit. To access, download the “2020-21 OB GYN Residency Applicant Spreadsheet” Google spreadsheet posted to the 2020–2021 “Residency Interview Spreadsheet” megathread on the r/medicalschool subreddit.

## Footnotes

1 “Simp” is a derogatory slang term to describe someone was overly self-sacrificing.
